# The fellow eye effect

**DOI:** 10.1038/s41433-026-04517-x

**Published:** 2026-05-07

**Authors:** Hashem Abu Serhan

**Affiliations:** Department of Ophthalmology, Hamad Medical Corporation, Doha, Qatar

**Keywords:** Retinal diseases, Education, Autoimmunity

Ophthalmology recognises that many ocular conditions exhibit bilateral tendencies, even when they initially present asymmetrically. Growing clinical and experimental evidence supports the concept of the “fellow eye effect,” whereby pathology or therapeutic interventions in one eye can influence the contralateral eye. This phenomenon spans multiple subspecialties, including glaucoma, retina, uveitis, and neuro-ophthalmology, and reflects a complex interplay of systemic, immunologic, and neurogenic mechanisms.

One of the most classic examples is sympathetic ophthalmia, a bilateral granulomatous panuveitis that develops after penetrating trauma or surgery to one eye. Despite the inciting event being unilateral, the fellow eye becomes inflamed due to loss of ocular immune privilege and subsequent autoimmune sensitisation to previously sequestered antigens [1]. This condition remains a cornerstone example of how the eye cannot be considered immunologically isolated, underscoring the systemic consequences of local injury.

In glaucoma, the fellow eye effect is more nuanced but equally important. Patients with unilateral angle recession glaucoma following trauma often exhibit an increased risk of glaucoma in the contralateral eye, reported to be as high as 50% [2]. Importantly, this risk does not arise from trauma itself but rather reflects an underlying bilateral susceptibility to glaucomatous optic neuropathy. Similarly, topical antiglaucoma medications, particularly beta-blockers such as timolol, demonstrate measurable intraocular pressure (IOP) reduction in the untreated fellow eye. This crossover effect is attributed to systemic absorption via the nasolacrimal system, leading to plasma drug levels sufficient to affect aqueous humour dynamics bilaterally [3].

Retinal diseases provide some of the most compelling and clinically relevant examples. Intravitreal anti–vascular endothelial growth factor (anti-VEGF) agents, including bevacizumab and aflibercept, have been repeatedly shown to exert effects in the contralateral eye [4, 5]. Patients receiving unilateral injections for diabetic macular oedema, neovascular age-related macular degeneration, or retinal vein occlusion may demonstrate anatomical or functional improvement in the untreated eye. Pharmacokinetic studies have confirmed that these agents enter the systemic circulation and reduce circulating VEGF levels, thereby influencing both eyes [6]. This phenomenon is particularly relevant when considering systemic safety and bilateral disease control.

Laser-based interventions also exhibit similar patterns. Panretinal photocoagulation (PRP), used in proliferative diabetic retinopathy, has been associated with reductions in circulating VEGF levels, occasionally leading to stabilisation or improvement in the fellow eye [7]. Although less consistent than anti-VEGF therapy, this observation supports the concept that intraocular treatments may have systemic biological consequences.

Surgical interventions further challenge the notion of ocular independence. Several studies have reported a reduction in IOP in the fellow eye following unilateral cataract surgery [8]. While the mechanism remains incompletely understood, proposed explanations include neurohumoral regulation of aqueous production and changes in central autonomic pathways. Similar observations have been described following trabeculectomy and minimally invasive glaucoma surgery (MIGS), although results are variable and may be influenced by behavioural factors such as improved medication adherence [9]. Not only that, in the surgical context, the fellow eye effect is particularly relevant. Clinical experience consistently shows that the second eye often mirrors the response of the first eye in terms of healing, inflammation, and overall surgical outcomes. Observations from the initial procedure, such as tissue behaviour, intraoperative challenges, and postoperative response, can therefore inform surgical planning, technique refinement, and risk stratification for the fellow eye, ultimately contributing to improved patient outcomes [8].

Neuro-ophthalmologic and visual system plasticity provide additional insight into bilateral interactions. In amblyopia therapy, occlusion of the dominant eye leads to functional improvement in the amblyopic eye through cortical reorganisation. Conversely, prolonged occlusion may induce “reverse amblyopia” in the patched eye, highlighting the dynamic and competitive nature of binocular cortical processing [10]. Likewise, fixation switch diplopia following unilateral visual rehabilitation illustrates how central adaptation can be disrupted when visual input is altered in one eye [10].

Other bilateral tendencies reflect systemic or choroidal dysregulation. Central serous chorioretinopathy (CSCR), although often clinically asymmetric, frequently demonstrates subclinical involvement in the fellow eye. Enhanced depth imaging and angiographic studies have revealed bilateral choroidal thickening and hyperpermeability, suggesting a systemic predisposition rather than a purely localised disorder [11]. Similarly, uveitic conditions initially presenting in one eye may later involve the fellow eye, reflecting underlying immune dysregulation [12].

The mechanisms underlying the fellow eye effect can be broadly categorised into several domains. Systemic absorption plays a central role in pharmacologic crossover, particularly for anti-VEGF agents and topical medications [3–5]. Immune-mediated processes are critical in conditions such as sympathetic ophthalmia, where antigen exposure triggers a bilateral response [1]. Neurogenic pathways may contribute to IOP regulation and retinal signalling, mediated through central autonomic networks and the optic chiasm [8]. Endocrine and cytokine-mediated effects, including circulating VEGF and inflammatory mediators, further support bilateral interactions [7]. Additionally, genetic predisposition explains why certain diseases, such as glaucoma and CSCR, often manifest asymmetrically despite being inherently bilateral [2, 11]. Finally, behavioural factors, including improved treatment adherence following intervention in one eye, may create the illusion of a biological fellow eye effect [9].

From a clinical perspective, recognising the fellow eye effect has important implications. First, it underscores the need for careful monitoring of both eyes, even in apparently unilateral disease. Second, it raises considerations regarding systemic exposure and safety, particularly with repeated intravitreal therapies. Third, it opens avenues for therapeutic innovation, where unilateral treatment strategies may confer bilateral benefit. Conversely, it also highlights potential risks, as systemic effects may lead to unintended consequences in the fellow eye.

In conclusion, the fellow eye effect underscores the inherently interconnected and often bilateral nature of ocular disease. As our understanding of these interactions evolves, clinicians should adopt a more holistic approach, recognising that intervention in one eye may have meaningful consequences for the other. Importantly, clinical and surgical experience in the first eye often provides valuable insights that can guide management of the fellow eye. Future research should aim to better characterise these mechanisms, quantify their clinical impact, and harness this phenomenon to optimise bilateral disease management.

## Data Availability

Data sharing not applicable to this article as no datasets were generated or analysed during the current study.
